# Dichloridotetra­kis­(diniconazole)cobalt(II)

**DOI:** 10.1107/S1600536811031291

**Published:** 2011-08-11

**Authors:** Chang-Xiang Liu, Xiu-Ying Song, Qian Liu, Xu-Liang Nie, Shi-He Wen

**Affiliations:** a College of Sciences, Jiangxi Agricultural University, Nanchang 330045, People’s Republic of China

## Abstract

In the crystal structure of the title compound, [CoCl_2_(C_15_H_17_Cl_2_N_3_O)_4_], the Co^II^ cation lies on an inversion center and has a slightly distorted octa­hedral coordination geometry. The equatorial positions are occupied by four N atoms from four diniconazole [systematic name: (*E*)-(*RS*)-1-(2,4-dichloro­phen­yl)-4,4-dimethyl-2-(1*H*-1,2,4-triazol-1-yl)pent-1-en-3-ol] ligands. The axial sites are occupied by two Cl^−^ anions. In the two independent organic ligands, the triazole ring is oriented at dihedral angles of 18.28 (14) and 32.15 (14)° with respect to the dichloro­phenyl ring. Inter­molecular O—H⋯Cl hydrogen bonds consolidate the crystal packing.

## Related literature

For background to the use of diniconazole as a fungicide, see: Sumitomo Chemical (1984 ▶); Huang *et al.* (2003 ▶). For further synthetic details, see: Fu (2002 ▶); Xia *et al.* (2001 ▶). For similar structures, see: Gao *et al.* (2001 ▶). For our previous work based on diniconazole, see: Xiong *et al.* (2010 ▶).
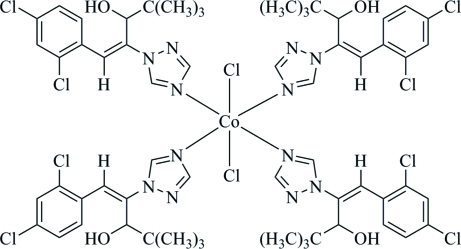


## Experimental

### 

#### Crystal data

[CoCl_2_(C_15_H_17_Cl_2_N_3_O)_4_]
*M* *_r_* = 1434.69
Triclinic, 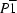

*a* = 8.800 (2) Å
*b* = 13.729 (4) Å
*c* = 15.145 (4) Å
α = 90.918 (3)°
β = 98.560 (3)°
γ = 106.775 (3)°
*V* = 1729.0 (8) Å^3^
*Z* = 1
Mo *K*α radiation
μ = 0.69 mm^−1^
*T* = 296 K
0.25 × 0.21 × 0.13 mm

#### Data collection

Bruker APEXII CCD diffractometer
Absorption correction: multi-scan (*SADABS*; Sheldrick, 2007 ▶) *T*_min_ = 0.847, *T*_max_ = 0.916
12895 measured reflections
6356 independent reflections
4764 reflections with *I* > 2σ(*I*)
*R*_int_ = 0.027

#### Refinement

*R*[*F* ^2^ > 2σ(*F* ^2^)] = 0.040
*wR*(*F* ^2^) = 0.098
*S* = 1.02
6356 reflections
402 parameters
70 restraints
H-atom parameters constrained
Δρ_max_ = 0.58 e Å^−3^
Δρ_min_ = −0.53 e Å^−3^

### 

Data collection: *APEX2* (Bruker, 2007 ▶); cell refinement: *SAINT* (Bruker, 2007 ▶); data reduction: *SAINT*; program(s) used to solve structure: *SHELXTL* (Sheldrick, 2008 ▶); program(s) used to refine structure: *SHELXTL*; molecular graphics: *SHELXTL*; software used to prepare material for publication: *SHELXTL*.

## Supplementary Material

Crystal structure: contains datablock(s) I, global. DOI: 10.1107/S1600536811031291/xu5277sup1.cif

Structure factors: contains datablock(s) I. DOI: 10.1107/S1600536811031291/xu5277Isup2.hkl

Additional supplementary materials:  crystallographic information; 3D view; checkCIF report

## Figures and Tables

**Table 1:** Hydrogen-bond geometry (Å, °)

*D*—H⋯*A*	*D*—H	H⋯*A*	*D*⋯*A*	*D*—H⋯*A*
O1—H1⋯Cl5^i^	0.82	2.35	3.136 (2)	161
O2—H2*A*⋯Cl5^i^	0.82	2.32	3.128 (2)	169

Symmetry code: (i) 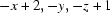.

**Fig. 1.**
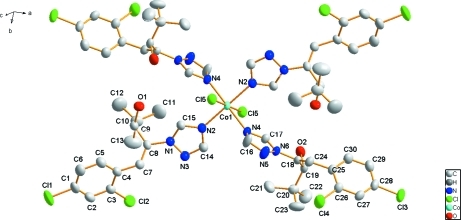
The molecular structure of (I), showing the atom-labelling scheme and displacement ellipsoids at the 40% probability level.

**Fig. 2.**
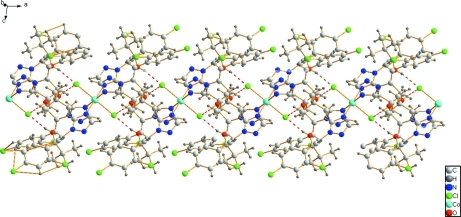
The crystal packing of (I), showing a hydrogen-bonded three- dimensional framework; H-bonds are shown as dashed lines.

## supplementary crystallographic information

### Comment

Diniconazole [(*E*)-(*RS*)-1-(2,4-dichlorophenyl)-4,4-dimethyl-2- (1*H*-1,2,4-triazol-1-yl)-pent-1-en-3-ol] is a highly active triazole fungicide(Sumitomo Chemical, 1984). It is widely used for control of a broad range of fungal diseases in many crops, such as corn, wheat, peanut, grape and apple (Huang *et al.*, 2003. Because of its strong antimicrobial activities and its wide applications, the synthesis of diniconazole(Fu *et al.*, 2002; Xia *et al.*, 2001) and its salts(Gao *et al.*, 2001) have attracted much attention. Recently, our group have reported the crystal structure of diniconazole(Xiong *et al.*, 2010). In this paper, we report the synthesis and crystal structure of a new cobalt(II) complex, (I), incorporating diniconazole.

The asymmetric unit of the title compound, [Co(C_15_H_17_Cl_2_N_3_O) _4_Cl_2_], consists of one cobalt(II) ion, two diniconazole ligands and one coordinated Cl atom. The Co atom lies on an inversion center and has a slightly distorted octahedral geometry. The equatorial positions are occupied by four N atoms from four (E)-(*RS*)-1- (2,4-dichlorophenyl)-4,4-dimethyl-2-(1*H*-1,2,4-triazol-1-yl)-pent -1-en-3-ol ligands. The axial sites are occupied by two Cl atoms (Fig. 1). The Co—N distance are 2.123 (3) and 2.147 (3) Å and Co—Cl is 2.5222 (9) Å. In the crystal packing, intermolecular O—H···Cl hydrogen bonds(Table 1) link the molecules into chains along the *a* axis (Fig. 2). The structure of the title compound is isostructural to previously reported zinc (II) complexe constructed by Zn (II) and diniconazole (Gao *et al.*, 2001).

### Experimental

CoCl_2_.6H_2_O (0.036 g, 0.1 mmol) was dissolved in ethanol (10 ml), and diniconazole (0.063 g, 0.2 mmol) was dissolved in ethanol (10 ml). The CoCl_2_ solution was added to the diniconazole solution slowly under stirring. The mixture were filtered after stirring for 1 h. Crystals suitable for X-ray analysis were obtained by slow concentration of an ethanol solution.

### Refinement

All H atoms were included in calculated positions and refined as riding atoms, with C—H = 0.93–0.96 Å, O—H = 0.82 Å, with *U*_iso_(H) = 1.5*U*_eq_(C) for methyl H and hydroxy atoms and 1.2*U*_eq_(C) for the others.

### Crystal data

**Table:**

[CoCl_2_(C_15_H_17_Cl_2_N_3_O)_4_]	*Z* = 1
*M**_r_* = 1434.69	*F*(000) = 741
Triclinic, *P*1	*D*_x_ = 1.378 Mg m^−^^3^
Hall symbol: -P 1	Mo *K*α radiation, λ = 0.71073 Å
*a* = 8.800 (2) Å	Cell parameters from 5326 reflections
*b* = 13.729 (4) Å	θ = 2.5–27.7°
*c* = 15.145 (4) Å	µ = 0.69 mm^−^^1^
α = 90.918 (3)°	*T* = 296 K
β = 98.560 (3)°	Block, red
γ = 106.775 (3)°	0.25 × 0.21 × 0.13 mm
*V* = 1729.0 (8) Å^3^	

### Data collection

**Table:**

Bruker APEXII CCD diffractometer	6356 independent reflections
Radiation source: fine-focus sealed tube	4764 reflections with *I* > 2σ(*I*)
graphite	*R*_int_ = 0.027
φ and ω scans	θ_max_ = 25.5°, θ_min_ = 2.5°
Absorption correction: multi-scan (*SADABS*; Sheldrick, 2007)	*h* = −10→10
*T*_min_ = 0.847, *T*_max_ = 0.916	*k* = −16→16
12895 measured reflections	*l* = −18→16

### Refinement

**Table:**

Refinement on *F*^2^	Primary atom site location: structure-invariant direct methods
Least-squares matrix: full	Secondary atom site location: difference Fourier map
*R*[*F*^2^ > 2σ(*F*^2^)] = 0.040	Hydrogen site location: inferred from neighbouring sites
*wR*(*F*^2^) = 0.098	H-atom parameters constrained
*S* = 1.02	*w* = 1/[σ^2^(*F*_o_^2^) + (0.0391*P*)^2^ + 0.9781*P*] where *P* = (*F*_o_^2^ + 2*F*_c_^2^)/3
6356 reflections	(Δ/σ)_max_ < 0.001
402 parameters	Δρ_max_ = 0.58 e Å^−^^3^
70 restraints	Δρ_min_ = −0.53 e Å^−^^3^

### Special details

**Table:**

Geometry. All e.s.d.'s (except the e.s.d. in the dihedral angle between two l.s. planes) are estimated using the full covariance matrix. The cell e.s.d.'s are taken into account individually in the estimation of e.s.d.'s in distances, angles and torsion angles; correlations between e.s.d.'s in cell parameters are only used when they are defined by crystal symmetry. An approximate (isotropic) treatment of cell e.s.d.'s is used for estimating e.s.d.'s involving l.s. planes.
Refinement. Refinement of *F*^2^ against ALL reflections. The weighted *R*-factor *wR* and goodness of fit *S* are based on *F*^2^, conventional *R*-factors *R* are based on *F*, with *F* set to zero for negative *F*^2^. The threshold expression of *F*^2^ > σ(*F*^2^) is used only for calculating *R*-factors(gt) *etc*. and is not relevant to the choice of reflections for refinement. *R*-factors based on *F*^2^ are statistically about twice as large as those based on *F*, and *R*- factors based on ALL data will be even larger.

### Fractional atomic coordinates and isotropic or equivalent isotropic displacement parameters (Å^2^)

**Table:**

	*x*	*y*	*z*	*U*_iso_*/*U*_eq_	
C1	1.0494 (4)	−0.3225 (2)	0.0361 (2)	0.0497 (8)	
C2	0.8863 (4)	−0.3484 (2)	0.0079 (2)	0.0489 (7)	
H2	0.8396	−0.3873	−0.0453	0.059*	
C3	0.7935 (3)	−0.3154 (2)	0.06012 (18)	0.0409 (7)	
C4	0.8601 (3)	−0.2571 (2)	0.13991 (17)	0.0354 (6)	
C5	1.0255 (3)	−0.2327 (2)	0.16483 (19)	0.0431 (7)	
H5	1.0734	−0.1933	0.2177	0.052*	
C6	1.1210 (4)	−0.2648 (2)	0.1137 (2)	0.0481 (7)	
H6	1.2317	−0.2477	0.1316	0.058*	
C7	0.7567 (3)	−0.2295 (2)	0.19802 (17)	0.0367 (6)	
H7	0.6785	−0.2831	0.2166	0.044*	
C8	0.7633 (3)	−0.1368 (2)	0.22641 (16)	0.0311 (6)	
C9	0.8686 (3)	−0.0379 (2)	0.19926 (17)	0.0362 (6)	
H9	0.9536	−0.0539	0.1721	0.043*	
C10	0.7825 (4)	0.0182 (2)	0.13025 (19)	0.0497 (8)	
C11	0.6677 (4)	0.0643 (3)	0.1702 (3)	0.0714 (10)	
H11A	0.7229	0.1041	0.2244	0.107*	
H11B	0.5777	0.0107	0.1831	0.107*	
H11C	0.6303	0.1072	0.1282	0.107*	
C12	0.9126 (5)	0.1039 (3)	0.0983 (3)	0.0819 (12)	
H12A	0.9661	0.1530	0.1472	0.123*	
H12B	0.8641	0.1365	0.0511	0.123*	
H12C	0.9893	0.0762	0.0764	0.123*	
C13	0.6918 (5)	−0.0564 (3)	0.0503 (2)	0.0814 (12)	
H13A	0.6532	−0.0197	0.0029	0.122*	
H13B	0.6024	−0.1063	0.0683	0.122*	
H13C	0.7629	−0.0899	0.0296	0.122*	
N3	0.4927 (3)	−0.18374 (18)	0.26583 (15)	0.0424 (4)	
C15	0.6774 (3)	−0.0721 (2)	0.36012 (17)	0.0359 (4)	
H15	0.7771	−0.0294	0.3865	0.043*	
C16	0.6166 (3)	0.2063 (2)	0.4043 (2)	0.0490 (8)	
H16	0.5121	0.2104	0.3907	0.059*	
C17	0.8114 (3)	0.15788 (19)	0.45944 (17)	0.0330 (6)	
H17	0.8764	0.1235	0.4915	0.040*	
C18	1.0204 (3)	0.27636 (18)	0.38500 (17)	0.0305 (6)	
C19	1.1615 (3)	0.27996 (19)	0.45688 (17)	0.0339 (6)	
H19	1.2570	0.2944	0.4276	0.041*	
C20	1.1966 (3)	0.3632 (2)	0.53304 (19)	0.0434 (7)	
C21	1.0705 (4)	0.3406 (3)	0.5948 (2)	0.0609 (9)	
H21A	1.0569	0.2734	0.6154	0.091*	
H21B	0.9701	0.3448	0.5628	0.091*	
H21C	1.1050	0.3896	0.6452	0.091*	
C22	1.3596 (4)	0.3668 (3)	0.5869 (2)	0.0714 (10)	
H22A	1.3883	0.4191	0.6343	0.107*	
H22B	1.4397	0.3814	0.5483	0.107*	
H22C	1.3532	0.3021	0.6117	0.107*	
C23	1.2078 (5)	0.4660 (2)	0.4928 (2)	0.0730 (11)	
H23A	1.2391	0.5189	0.5399	0.110*	
H23B	1.1050	0.4644	0.4599	0.110*	
H23C	1.2863	0.4792	0.4533	0.110*	
C24	1.0297 (3)	0.3108 (2)	0.30455 (17)	0.0358 (6)	
H24	0.9347	0.3106	0.2681	0.043*	
C25	1.1823 (3)	0.3496 (2)	0.26884 (17)	0.0349 (6)	
C26	1.2355 (3)	0.4487 (2)	0.24285 (17)	0.0373 (6)	
C27	1.3818 (3)	0.4862 (2)	0.21383 (18)	0.0410 (7)	
H27	1.4151	0.5529	0.1970	0.049*	
C28	1.4770 (3)	0.4232 (2)	0.21036 (17)	0.0406 (7)	
C29	1.4252 (3)	0.3228 (2)	0.23034 (19)	0.0455 (7)	
H29	1.4881	0.2798	0.2246	0.055*	
C30	1.2782 (3)	0.2868 (2)	0.25912 (18)	0.0432 (7)	
H30	1.2426	0.2188	0.2723	0.052*	
Cl1	1.16567 (13)	−0.36529 (9)	−0.02980 (7)	0.0868 (3)	
Cl2	0.58804 (9)	−0.34674 (7)	0.02462 (6)	0.0630 (2)	
Cl3	1.66675 (9)	0.47063 (7)	0.18178 (6)	0.0626 (2)	
Cl4	1.11859 (10)	0.52998 (7)	0.24962 (6)	0.0644 (2)	
Cl5	0.73853 (7)	−0.02334 (5)	0.60279 (5)	0.04108 (17)	
Co1	0.5000	0.0000	0.5000	0.02659 (13)	
N1	0.6509 (2)	−0.12807 (17)	0.28403 (14)	0.0363 (4)	
N2	0.5432 (2)	−0.08524 (16)	0.39304 (14)	0.0367 (4)	
C14	0.4351 (3)	−0.1547 (2)	0.33316 (18)	0.0417 (4)	
H14	0.3274	−0.1800	0.3394	0.050*	
N4	0.6547 (2)	0.13667 (16)	0.45758 (14)	0.0335 (5)	
N5	0.7373 (3)	0.26797 (19)	0.37297 (18)	0.0511 (7)	
N6	0.8632 (2)	0.23515 (15)	0.40909 (14)	0.0319 (5)	
O1	0.9452 (2)	0.03136 (14)	0.27492 (13)	0.0439 (5)	
H1	1.0267	0.0181	0.2980	0.066*	
O2	1.1451 (2)	0.18421 (14)	0.49524 (13)	0.0437 (5)	
H2A	1.1807	0.1485	0.4650	0.066*	

### Atomic displacement parameters (Å^2^)

**Table:**

	*U*^11^	*U*^22^	*U*^33^	*U*^12^	*U*^13^	*U*^23^
C1	0.062 (2)	0.0462 (18)	0.0542 (19)	0.0276 (15)	0.0284 (16)	0.0082 (15)
C2	0.065 (2)	0.0433 (17)	0.0407 (16)	0.0158 (15)	0.0172 (15)	−0.0033 (13)
C3	0.0473 (16)	0.0380 (16)	0.0386 (16)	0.0117 (13)	0.0122 (13)	0.0010 (12)
C4	0.0451 (15)	0.0346 (15)	0.0301 (14)	0.0150 (12)	0.0106 (12)	0.0042 (11)
C5	0.0500 (17)	0.0476 (17)	0.0355 (15)	0.0212 (14)	0.0052 (13)	0.0014 (13)
C6	0.0470 (17)	0.0503 (18)	0.0540 (19)	0.0226 (14)	0.0128 (15)	0.0096 (15)
C7	0.0412 (15)	0.0382 (15)	0.0307 (14)	0.0095 (12)	0.0103 (12)	0.0022 (12)
C8	0.0315 (13)	0.0412 (15)	0.0228 (12)	0.0142 (11)	0.0040 (10)	0.0001 (11)
C9	0.0371 (14)	0.0386 (15)	0.0346 (14)	0.0125 (12)	0.0092 (12)	−0.0005 (12)
C10	0.064 (2)	0.0461 (18)	0.0389 (17)	0.0189 (15)	0.0023 (15)	0.0089 (14)
C11	0.076 (2)	0.075 (3)	0.076 (2)	0.045 (2)	0.003 (2)	0.016 (2)
C12	0.109 (3)	0.063 (2)	0.076 (3)	0.019 (2)	0.028 (2)	0.031 (2)
C13	0.116 (3)	0.074 (3)	0.046 (2)	0.031 (2)	−0.021 (2)	0.0038 (18)
N3	0.0373 (6)	0.0467 (7)	0.0409 (6)	0.0086 (5)	0.0078 (5)	−0.0036 (5)
C15	0.0346 (6)	0.0391 (7)	0.0348 (6)	0.0112 (5)	0.0073 (5)	−0.0007 (5)
C16	0.0280 (14)	0.0559 (19)	0.069 (2)	0.0161 (13)	0.0152 (14)	0.0278 (16)
C17	0.0295 (13)	0.0343 (14)	0.0348 (14)	0.0074 (11)	0.0075 (11)	0.0082 (11)
C18	0.0279 (12)	0.0274 (13)	0.0369 (14)	0.0063 (10)	0.0102 (11)	0.0051 (11)
C19	0.0288 (13)	0.0374 (15)	0.0384 (15)	0.0106 (11)	0.0124 (11)	0.0079 (12)
C20	0.0435 (16)	0.0433 (17)	0.0398 (16)	0.0090 (13)	0.0030 (13)	0.0003 (13)
C21	0.065 (2)	0.072 (2)	0.0461 (18)	0.0189 (18)	0.0133 (16)	−0.0139 (16)
C22	0.053 (2)	0.093 (3)	0.055 (2)	0.0075 (19)	−0.0067 (17)	−0.0029 (19)
C23	0.097 (3)	0.0367 (19)	0.074 (2)	0.0077 (18)	0.003 (2)	−0.0073 (17)
C24	0.0313 (13)	0.0375 (15)	0.0368 (15)	0.0056 (11)	0.0077 (11)	0.0090 (12)
C25	0.0324 (13)	0.0406 (16)	0.0297 (14)	0.0063 (12)	0.0075 (11)	0.0061 (12)
C26	0.0359 (14)	0.0423 (16)	0.0337 (14)	0.0103 (12)	0.0074 (12)	0.0082 (12)
C27	0.0389 (15)	0.0401 (16)	0.0393 (15)	0.0019 (13)	0.0105 (12)	0.0087 (13)
C28	0.0319 (14)	0.0547 (18)	0.0317 (14)	0.0048 (13)	0.0104 (12)	0.0033 (13)
C29	0.0462 (16)	0.0555 (19)	0.0415 (16)	0.0216 (14)	0.0148 (13)	0.0054 (14)
C30	0.0489 (16)	0.0409 (16)	0.0423 (16)	0.0119 (13)	0.0165 (13)	0.0103 (13)
Cl1	0.0909 (7)	0.0960 (8)	0.0971 (7)	0.0481 (6)	0.0490 (6)	−0.0096 (6)
Cl2	0.0487 (4)	0.0748 (6)	0.0564 (5)	0.0078 (4)	0.0031 (4)	−0.0177 (4)
Cl3	0.0381 (4)	0.0823 (6)	0.0635 (5)	0.0041 (4)	0.0239 (4)	0.0016 (4)
Cl4	0.0627 (5)	0.0574 (5)	0.0889 (6)	0.0315 (4)	0.0316 (5)	0.0308 (5)
Cl5	0.0340 (3)	0.0487 (4)	0.0436 (4)	0.0183 (3)	0.0032 (3)	0.0068 (3)
Co1	0.0233 (2)	0.0310 (3)	0.0276 (3)	0.00848 (19)	0.00932 (19)	0.0044 (2)
N1	0.0343 (6)	0.0401 (6)	0.0350 (6)	0.0107 (5)	0.0075 (5)	−0.0014 (5)
N2	0.0346 (6)	0.0403 (6)	0.0362 (6)	0.0117 (5)	0.0078 (5)	0.0000 (5)
C14	0.0371 (6)	0.0458 (7)	0.0410 (7)	0.0098 (6)	0.0079 (5)	−0.0021 (6)
N4	0.0295 (11)	0.0347 (12)	0.0377 (12)	0.0089 (9)	0.0111 (9)	0.0078 (10)
N5	0.0357 (13)	0.0525 (16)	0.0721 (18)	0.0185 (11)	0.0166 (12)	0.0324 (14)
N6	0.0273 (10)	0.0316 (12)	0.0383 (12)	0.0088 (9)	0.0095 (9)	0.0095 (10)
O1	0.0360 (10)	0.0456 (12)	0.0459 (12)	0.0106 (9)	−0.0032 (9)	−0.0067 (9)
O2	0.0482 (11)	0.0436 (11)	0.0501 (12)	0.0257 (9)	0.0159 (9)	0.0145 (9)

### Geometric parameters (Å, °)

**Table:**

C1—C6	1.367 (4)	C18—C24	1.319 (3)
C1—C2	1.373 (4)	C18—N6	1.439 (3)
C1—Cl1	1.737 (3)	C18—C19	1.513 (3)
C2—C3	1.376 (4)	C19—O2	1.424 (3)
C2—H2	0.9300	C19—C20	1.543 (4)
C3—C4	1.389 (4)	C19—H19	0.9800
C3—Cl2	1.731 (3)	C20—C21	1.525 (4)
C4—C5	1.387 (4)	C20—C22	1.527 (4)
C4—C7	1.474 (3)	C20—C23	1.529 (4)
C5—C6	1.378 (4)	C21—H21A	0.9600
C5—H5	0.9300	C21—H21B	0.9600
C6—H6	0.9300	C21—H21C	0.9600
C7—C8	1.320 (4)	C22—H22A	0.9600
C7—H7	0.9300	C22—H22B	0.9600
C8—N1	1.441 (3)	C22—H22C	0.9600
C8—C9	1.509 (4)	C23—H23A	0.9600
C9—O1	1.427 (3)	C23—H23B	0.9600
C9—C10	1.544 (4)	C23—H23C	0.9600
C9—H9	0.9800	C24—C25	1.481 (3)
C10—C11	1.525 (4)	C24—H24	0.9300
C10—C12	1.529 (5)	C25—C26	1.389 (4)
C10—C13	1.534 (4)	C25—C30	1.389 (4)
C11—H11A	0.9600	C26—C27	1.382 (4)
C11—H11B	0.9600	C26—Cl4	1.733 (3)
C11—H11C	0.9600	C27—C28	1.373 (4)
C12—H12A	0.9600	C27—H27	0.9300
C12—H12B	0.9600	C28—C29	1.374 (4)
C12—H12C	0.9600	C28—Cl3	1.731 (3)
C13—H13A	0.9600	C29—C30	1.383 (4)
C13—H13B	0.9600	C29—H29	0.9300
C13—H13C	0.9600	C30—H30	0.9300
N3—C14	1.309 (3)	Cl5—Co1	2.5227 (8)
N3—N1	1.365 (3)	Co1—N2	2.126 (2)
C15—N2	1.315 (3)	Co1—N2^i^	2.126 (2)
C15—N1	1.327 (3)	Co1—N4^i^	2.147 (2)
C15—H15	0.9300	Co1—N4	2.147 (2)
C16—N5	1.309 (3)	Co1—Cl5^i^	2.5227 (8)
C16—N4	1.344 (3)	N2—C14	1.352 (3)
C16—H16	0.9300	C14—H14	0.9300
C17—N4	1.321 (3)	N5—N6	1.359 (3)
C17—N6	1.328 (3)	O1—H1	0.8200
C17—H17	0.9300	O2—H2A	0.8200
			
C6—C1—C2	121.9 (3)	C21—C20—C19	112.8 (2)
C6—C1—Cl1	119.8 (2)	C22—C20—C19	106.4 (3)
C2—C1—Cl1	118.3 (2)	C23—C20—C19	109.3 (2)
C1—C2—C3	118.5 (3)	C20—C21—H21A	109.5
C1—C2—H2	120.7	C20—C21—H21B	109.5
C3—C2—H2	120.7	H21A—C21—H21B	109.5
C2—C3—C4	121.9 (3)	C20—C21—H21C	109.5
C2—C3—Cl2	118.8 (2)	H21A—C21—H21C	109.5
C4—C3—Cl2	119.3 (2)	H21B—C21—H21C	109.5
C5—C4—C3	117.1 (2)	C20—C22—H22A	109.5
C5—C4—C7	122.0 (2)	C20—C22—H22B	109.5
C3—C4—C7	120.7 (2)	H22A—C22—H22B	109.5
C6—C5—C4	122.0 (3)	C20—C22—H22C	109.5
C6—C5—H5	119.0	H22A—C22—H22C	109.5
C4—C5—H5	119.0	H22B—C22—H22C	109.5
C1—C6—C5	118.5 (3)	C20—C23—H23A	109.5
C1—C6—H6	120.7	C20—C23—H23B	109.5
C5—C6—H6	120.7	H23A—C23—H23B	109.5
C8—C7—C4	126.8 (2)	C20—C23—H23C	109.5
C8—C7—H7	116.6	H23A—C23—H23C	109.5
C4—C7—H7	116.6	H23B—C23—H23C	109.5
C7—C8—N1	117.2 (2)	C18—C24—C25	123.9 (2)
C7—C8—C9	126.6 (2)	C18—C24—H24	118.0
N1—C8—C9	116.1 (2)	C25—C24—H24	118.0
O1—C9—C8	111.5 (2)	C26—C25—C30	117.1 (2)
O1—C9—C10	108.0 (2)	C26—C25—C24	122.3 (2)
C8—C9—C10	115.1 (2)	C30—C25—C24	120.6 (2)
O1—C9—H9	107.3	C27—C26—C25	122.1 (3)
C8—C9—H9	107.3	C27—C26—Cl4	118.3 (2)
C10—C9—H9	107.3	C25—C26—Cl4	119.6 (2)
C11—C10—C12	108.8 (3)	C28—C27—C26	118.8 (3)
C11—C10—C13	110.4 (3)	C28—C27—H27	120.6
C12—C10—C13	108.9 (3)	C26—C27—H27	120.6
C11—C10—C9	112.4 (2)	C27—C28—C29	121.1 (2)
C12—C10—C9	107.2 (3)	C27—C28—Cl3	119.7 (2)
C13—C10—C9	109.1 (3)	C29—C28—Cl3	119.1 (2)
C10—C11—H11A	109.5	C28—C29—C30	119.0 (3)
C10—C11—H11B	109.5	C28—C29—H29	120.5
H11A—C11—H11B	109.5	C30—C29—H29	120.5
C10—C11—H11C	109.5	C29—C30—C25	121.7 (3)
H11A—C11—H11C	109.5	C29—C30—H30	119.2
H11B—C11—H11C	109.5	C25—C30—H30	119.2
C10—C12—H12A	109.5	N2—Co1—N2^i^	180.0
C10—C12—H12B	109.5	N2—Co1—N4^i^	90.22 (8)
H12A—C12—H12B	109.5	N2^i^—Co1—N4^i^	89.78 (8)
C10—C12—H12C	109.5	N2—Co1—N4	89.78 (8)
H12A—C12—H12C	109.5	N2^i^—Co1—N4	90.22 (8)
H12B—C12—H12C	109.5	N4^i^—Co1—N4	180.00 (8)
C10—C13—H13A	109.5	N2—Co1—Cl5^i^	88.23 (6)
C10—C13—H13B	109.5	N2^i^—Co1—Cl5^i^	91.77 (6)
H13A—C13—H13B	109.5	N4^i^—Co1—Cl5^i^	89.15 (6)
C10—C13—H13C	109.5	N4—Co1—Cl5^i^	90.85 (6)
H13A—C13—H13C	109.5	N2—Co1—Cl5	91.77 (6)
H13B—C13—H13C	109.5	N2^i^—Co1—Cl5	88.23 (6)
C14—N3—N1	102.1 (2)	N4^i^—Co1—Cl5	90.85 (6)
N2—C15—N1	110.7 (2)	N4—Co1—Cl5	89.15 (6)
N2—C15—H15	124.6	Cl5^i^—Co1—Cl5	180.00 (3)
N1—C15—H15	124.6	C15—N1—N3	109.4 (2)
N5—C16—N4	115.2 (2)	C15—N1—C8	129.1 (2)
N5—C16—H16	122.4	N3—N1—C8	121.5 (2)
N4—C16—H16	122.4	C15—N2—C14	102.5 (2)
N4—C17—N6	110.5 (2)	C15—N2—Co1	128.29 (18)
N4—C17—H17	124.8	C14—N2—Co1	128.41 (17)
N6—C17—H17	124.8	N3—C14—N2	115.2 (2)
C24—C18—N6	118.0 (2)	N3—C14—H14	122.4
C24—C18—C19	125.8 (2)	N2—C14—H14	122.4
N6—C18—C19	116.1 (2)	C17—N4—C16	102.6 (2)
O2—C19—C18	111.8 (2)	C17—N4—Co1	126.15 (17)
O2—C19—C20	108.6 (2)	C16—N4—Co1	129.29 (17)
C18—C19—C20	115.3 (2)	C16—N5—N6	102.5 (2)
O2—C19—H19	106.9	C17—N6—N5	109.3 (2)
C18—C19—H19	106.9	C17—N6—C18	129.1 (2)
C20—C19—H19	106.9	N5—N6—C18	121.3 (2)
C21—C20—C22	109.1 (3)	C9—O1—H1	109.5
C21—C20—C23	109.9 (3)	C19—O2—H2A	109.5
C22—C20—C23	109.3 (3)		
			
C6—C1—C2—C3	−0.4 (5)	Cl3—C28—C29—C30	175.6 (2)
Cl1—C1—C2—C3	179.5 (2)	C28—C29—C30—C25	−0.4 (4)
C1—C2—C3—C4	0.0 (4)	C26—C25—C30—C29	4.1 (4)
C1—C2—C3—Cl2	179.5 (2)	C24—C25—C30—C29	−176.6 (3)
C2—C3—C4—C5	0.4 (4)	N2—C15—N1—N3	−1.7 (3)
Cl2—C3—C4—C5	−179.1 (2)	N2—C15—N1—C8	−179.8 (2)
C2—C3—C4—C7	−175.2 (3)	C14—N3—N1—C15	1.1 (3)
Cl2—C3—C4—C7	5.3 (4)	C14—N3—N1—C8	179.4 (2)
C3—C4—C5—C6	−0.5 (4)	C7—C8—N1—C15	133.4 (3)
C7—C4—C5—C6	175.1 (3)	C9—C8—N1—C15	−50.9 (4)
C2—C1—C6—C5	0.4 (5)	C7—C8—N1—N3	−44.5 (3)
Cl1—C1—C6—C5	−179.5 (2)	C9—C8—N1—N3	131.1 (2)
C4—C5—C6—C1	0.0 (4)	N1—C15—N2—C14	1.5 (3)
C5—C4—C7—C8	60.7 (4)	N1—C15—N2—Co1	−168.98 (17)
C3—C4—C7—C8	−123.9 (3)	N2^i^—Co1—N2—C15	46 (100)
C4—C7—C8—N1	−179.2 (2)	N4^i^—Co1—N2—C15	−143.4 (2)
C4—C7—C8—C9	5.7 (4)	N4—Co1—N2—C15	36.6 (2)
C7—C8—C9—O1	−134.4 (3)	Cl5^i^—Co1—N2—C15	127.4 (2)
N1—C8—C9—O1	50.5 (3)	Cl5—Co1—N2—C15	−52.6 (2)
C7—C8—C9—C10	102.1 (3)	N2^i^—Co1—N2—C14	−122 (100)
N1—C8—C9—C10	−73.1 (3)	N4^i^—Co1—N2—C14	48.5 (2)
O1—C9—C10—C11	−54.3 (3)	N4—Co1—N2—C14	−131.5 (2)
C8—C9—C10—C11	71.1 (3)	Cl5^i^—Co1—N2—C14	−40.7 (2)
O1—C9—C10—C12	65.2 (3)	Cl5—Co1—N2—C14	139.3 (2)
C8—C9—C10—C12	−169.4 (3)	N1—N3—C14—N2	−0.1 (3)
O1—C9—C10—C13	−177.1 (3)	C15—N2—C14—N3	−0.8 (3)
C8—C9—C10—C13	−51.7 (3)	Co1—N2—C14—N3	169.63 (19)
C24—C18—C19—O2	−131.9 (3)	N6—C17—N4—C16	−0.7 (3)
N6—C18—C19—O2	50.7 (3)	N6—C17—N4—Co1	164.63 (16)
C24—C18—C19—C20	103.3 (3)	N5—C16—N4—C17	0.4 (4)
N6—C18—C19—C20	−74.0 (3)	N5—C16—N4—Co1	−164.3 (2)
O2—C19—C20—C21	−55.1 (3)	N2—Co1—N4—C17	−66.5 (2)
C18—C19—C20—C21	71.2 (3)	N2^i^—Co1—N4—C17	113.5 (2)
O2—C19—C20—C22	64.4 (3)	N4^i^—Co1—N4—C17	151 (100)
C18—C19—C20—C22	−169.2 (2)	Cl5^i^—Co1—N4—C17	−154.7 (2)
O2—C19—C20—C23	−177.7 (2)	Cl5—Co1—N4—C17	25.3 (2)
C18—C19—C20—C23	−51.4 (3)	N2—Co1—N4—C16	94.9 (2)
N6—C18—C24—C25	−176.3 (2)	N2^i^—Co1—N4—C16	−85.1 (2)
C19—C18—C24—C25	6.3 (4)	N4^i^—Co1—N4—C16	−48 (100)
C18—C24—C25—C26	−119.7 (3)	Cl5^i^—Co1—N4—C16	6.7 (2)
C18—C24—C25—C30	61.0 (4)	Cl5—Co1—N4—C16	−173.3 (2)
C30—C25—C26—C27	−4.0 (4)	N4—C16—N5—N6	0.1 (4)
C24—C25—C26—C27	176.7 (2)	N4—C17—N6—N5	0.8 (3)
C30—C25—C26—Cl4	177.9 (2)	N4—C17—N6—C18	−173.2 (2)
C24—C25—C26—Cl4	−1.5 (4)	C16—N5—N6—C17	−0.5 (3)
C25—C26—C27—C28	0.2 (4)	C16—N5—N6—C18	174.0 (2)
Cl4—C26—C27—C28	178.4 (2)	C24—C18—N6—C17	144.7 (3)
C26—C27—C28—C29	3.7 (4)	C19—C18—N6—C17	−37.7 (4)
C26—C27—C28—Cl3	−175.5 (2)	C24—C18—N6—N5	−28.7 (4)
C27—C28—C29—C30	−3.6 (4)	C19—C18—N6—N5	148.9 (2)

Symmetry codes: (i) −*x*+1, −*y*, −*z*+1.

### Hydrogen-bond geometry (Å, °)

**Table:**

*D*—H···*A*	*D*—H	H···*A*	*D*···*A*	*D*—H···*A*
O1—H1···Cl5^ii^	0.82	2.35	3.136 (2)	161.
O2—H2A···Cl5^ii^	0.82	2.32	3.128 (2)	169.

Symmetry codes: (ii) −*x*+2, −*y*, −*z*+1.
